# Supplementary material for: WordCommentsAnalyzer: A windows software tool for qualitative research
Source: F1000Res. 2018 Sep 4;7:536. Originally published 2018 May 3. [Version 2] doi: 10.12688/f1000research.14819.2 (PMC6137411; doi:10.12688/f1000research.14819.2)
Supplement: Supplementary file 3 [file f1000research-7-17614-s0002.tgz › b2a79496-4ada-47c4-9682-a99ce867836d.zip!/Eight Iranian Health Journals/The Journal of Toloo-e-Behdasht_29.docx]

© The Journal of Toloo-e-Behdasht. This work is licensed under CC BY 4.0 (https://creativecommons.org/licenses/by/4.0/).

Volume & Issue: دوره 12 - شماره 3

Title: جذب تولوئن هوا با استفاده از سلولز باکتریایی

http://tbj.ssu.ac.ir/article-1-696-fa.html

مقدمه: بافت شبکه ای یکسان و محکم در سلولز باکتریایی امکان استفاده از آن را به عنوان فیلتر و جاذب فراهم می نماید. در این تحقیق از سلولز باکتریایی حاصل از رشد و تکثیر باکتری استوباکتر گزیلینوم که قبلا در آزمایشگاه تولید شده و با تابش اشعه مایکروویو به صورت جامد متورم تبدیل شده بود به عنوان جاذب بخارات تولوئن از هوا استفاده گردید.   روش بررسی: برای اندازه گیری ظرفیت جذب، مقدار 6 گرم سلولز میکروبی در ستون شیشه ای با حجم 20 میلی لیتر قرار گرفت و غلظت ppm 150 از بخارات تولوئن با جریان هوای عبوری ml/min 2/0 در دمای 25 درجه سانتیگراد، از بستر جاذب عبور داده شد و همزمان مقدار تولوئن خروجی در زمانهای مختلف اندازه گیری گردید.   یافته ها: یک گرم سلولز میکروبی متورم و دانه بندی شده با مش20 می تواند نزدیک به 1میلی گرم ( mg 95/0) بخارات تولوئن را در طی مدت 13 دقیقه جذب نماید و به حالت اشباع کامل برسد. همچنین میزان 20 سانتی متر مکعب کربن اکتیو و سلولز میکروبی در شرایط یکسان به ترتیب پس از 70 دقیقه و 39 دقیقه به حالت اشباع رسیدند.   نتیجه گیری: هرچند که سلولز باکتریایی در حجم یکسان ظرفیت کمتری نسبت به کربن اکتیو دارد ولی با توجه به اینکه چگالی آن بسیار کمتر می باشد می توان گفت که توانایی وزنی سلولز باکتریایی بالاتر از کربن اکتیو در جذب بخارات تولوئن بوده است.

Title: نگرش مسئولین به میزان تاثیرعوامل آمیخته بازاریابی در بیمارستانهای شهر یزد

http://tbj.ssu.ac.ir/article-1-697-fa.html

مقدمه :با توجه به هزینه بری زیاد خدمات در بیمارستانها مخصوصا بیمارستانهای دولتی و ضرورت ساماندهی به امور اقتصادی آنها نیاز به شناسایی ومشخص شدن تاثیر هریک از عوامل موثر بر بازاریابی خدمات بهداشتی درمانی در بیمارستانها می باشد. در این مطالعه تاثیرعوامل بازاریابی خدمات ازنظر مشتریان داخلی بررسی شد.   روش بررسی :پژوهش حاضر از نوع پژوهش کاربردی و مقطعی است. تعداد نمونه 33نفر از مجموعه مدیران ارشد و میانی شامل مدیر داخلی، مدیر پرستاری، سوپروایزرهای آموزشی، مسئولین اداری مالی، آزمایشگاه، داروخانه و رادیولوژی بیمارستانهای شهر یزد را شامل می شود که از طریق سرشماری در پاییز1390مورد بررسی قرارگرفته است.ابزار گردآوری داده ها پرسشنامه و داده ها توسط نرم افزارهای آماری بویژه spss و statistica مورد تجزیه وتحلیل قرار گرفت.   یافته ها : بیشترین فراوانی (درصد) پاسخ مدیران در ارتباط بانقش آنها در بکارگیری عامل خدمت یا محصول دربیمارستانهای یزد در حد متوسط (2/72%) ومتعلق به مسئولین آزمایشگاه،عامل قیمت در حد کم (6/63%) ومتعلق به مسئولین اداری مالی،عامل توزیع در حد متوسط (50%) ومتعلق به مسئولین آزمایشگاه،عامل ارتقاء یا تبلیغ در حد خیلی کم (4/68%) ومتعلق به مسئولین اداری مالی، عامل مردم یا اشخاص در حد زیاد (3/73%) ومتعلق به مسئولین رادیولوژی،عامل شواهد فیزیکی در حد زیاد (6/47%) ومتعلق به مدیران بیمارستانها،عامل فرایند در حد متوسط (4/44%) ومتعلق به سوپروایزرهای آموزشی،عامل بهره وری وکیفیت در حد متوسط (7/66%) ومتعلق به مسئولین آزمایشگاه ارزیابی شده است.   نتیجه گیری: براساس نتایج حاصله از این پژوهش درصد بیشتری از مترونها اعتقاد به نقش خود در بکارگیری عوامل خدمت، مردم یا اشخاص،شواهد فیزیکی،فرایندو بهره وری وکیفیت داشتند. همچنین مسئولین رادیولوژی در بکارگیری عوامل توزیع وارتقاء،مسئولین داروخانه دربکارگیری عامل قیمت و مدیران بیمارستانها اعتقاد به نقش زیادومتوسط خود در بکار گیری عوامل موثر بازاریابی خدمات بهداشتی درمانی داشتند.

Title: بررسی میزان آگاهی، نگرش و عملکرد زنان خانه دار شهر یزد در زمینه بازیافت مواد زائد جامد

http://tbj.ssu.ac.ir/article-1-698-fa.html

مقدمه: امروزه بازیافت یکی از راه‌های نجات بشر از انبوه زباله‌هایی است که تولید می‌کند. در واقع بازیافت به معنای یک سیستم با هدف بهینه سازی مصرف منابع مختلف به منظور دستیابی به سود کلی بیشتر همراه با تولید مواد زائد کم‌تر است. هدف از انجام این پژوهش بررسی میزان آگاهی، نگرش و عملکرد و زنان خانه دار شهر یزد در زمینه بازیافت مواد زائد جامد بود.   روش بررسی: این یک مطالعه توصیفی- تحلیلی از نوع مقطعی بود و جامعه مورد پژوهش زنان خانه دار شهر یزد بودند که در یک نمونه 300 نفری به صورت خوشه‌ای تصادفی از پنج منطقه شهر شامل: شمال، جنوب، شرق، غرب، مرکز و از هر منطقه دو خوشه (هر خوشه شامل یک محله بود که 32 خانوار آن مورد بررسی قرار می‌گرفت) انتخاب گردیدند. ابزار گردآوری اطلاعات، پرسشنامه محقق ساخته دارای روایی و پایایی معتبر در چهار بخش جمعیت شناختی، آگاهی، نگرش و عملکرد می‌باشد که به صورت مصاحبه و خودگزارش دهی توسط دو کارشناس آموزش دیده تکمیل گردیدند.   یافته‌ها: میانگین سن شرکت کنندگان 21/15±84/41، 7/32% (98 نفر) دارای تحصیلات دیپلم، 88% (264 نفر) دارای وضعیت اقتصادی متوسط، 84% (252 نفر) متاهل، 3/50% (151 نفر) خانه دار، 3/83% (250 نفر) با وضعیت سکونت مالکیت بودند. میانگین نمره آگاهی، نگرش و عملکرد آنها به ترتیب برابر با 91/1±21/8،(از کل 14 امتیاز) 47/4±59/36 ( از کل 50 امتیاز) و 67/0±89/1 ( از کل 3 امتیاز) بود. بین سطح تحصیلات زنان با آگاهی (001/0 > p ) با نگرش (004/0 = p ) و بین شغل و آگاهی (001/0> p ) آن‌ها رابطه آماری معناداری وجود داشت. همچنین همبستگی معناداری بین میانگین نمره آگاهی و نگرش (283/0)، آگاهی و عملکرد (122/0) و عملکرد و نگرش (241/0) وجود داشت.   نتیجه گیری: این مطالعه نشان داد میزان آگاهی، نگرش و عملکرد زنان در سطح قابل قبولی قرار داشته اما با تاکید بیشتر بر جنبه‌های آموزشی خصوصا از طریق شهرداری‌ها می‌توان عملکرد بهتری از زنان را شاهد بود. همچنین با ایجاد برنامه‌های مشارکتی و تشویقی بین خانواده و شهرداری‌ها می‌توان در ارتقاء این فعالیت زیست محیطی گام‌هایی در خور توجه برداشت.

Title: ارزشیابی اثر بخشی دوره های آموزش بهداشت اصناف کشور مبتنی بر مدل کرک پاتریک

http://tbj.ssu.ac.ir/article-1-699-fa.html

مقدمه: با توجه به اهمیت بهداشت و سلامت جامعه و انتقال و شیوع بسیاری از بیماری‌ها از راه های مختلف از جمله دست اندرکاران تهیه، تولید و توزیع مواد غذایی و با توجه به برگزاری دوره های آموزش بهداشت اصناف طی دوازده سال گذشته در کشور و اهمیت ارزشیابی آن و اینکه مدل کرک پاتریک یکی از مهم‌ترین مدل ها برای ارزشیابی دوره های آموزشی می باشد لذا این مطالعه با هدف تعیین میزان اثر بخشی دوره های آموزش بهداشت اصناف کشور مبتنی بر مدل کرک پاتریک صورت گرفت.   روش بررسی: مطالعه‌ توصیفی حاضر از نوع مقطعی بود و در میان 900 نفر از کسبه دارای گواهینامه بهداشت اصناف انجام گرفت. نمونه ها به صورت تصادفی از پانزده استان انتخاب شدند و مورد مصاحبه قرار گرفتند. ابزار گردآوری اطلاعات پرسشنامه مبتنی بر مدل کرک پاتریک شامل: اطلاعات زمینه ای و جمعیت شناختی با هشت سوال، 35 سوال آگاهی، 5 گویه نگرش در خصوص اهمیت برگزاری و گذراندن دوره های آموزش بهداشت اصناف، 21 سوال عملکرد برحسب تعدادی از ماده های فرم آئین نامه مقررات بهداشتی اصناف و ده گویه رضایتمندی از نحوه برگزاری کلاس‌ها بود. داده‌ها توسط SPSS ویرایش هجدهم و با کمک آمار توصیفی و آزمون پارامتریک تحلیلی ضریب همبستگی پیرسون در سطح معناداری 05/0 مورد آزمون قرار گرفتند.   یافته‌ها: میانگین سن شرکت کنندگان 55/13 ± 03/36 سال بود. حداکثر امتیاز کسب شده و بالاترین فراوانی شرکت کنندگان در هر سطح ارزشی سازه ها به ترتیب؛ آگاهی (باز پاسخ) 18/67 درصد و 7/59 (خوب) درصد، آگاهی (چند گزینه ای) 77/62 و 8/64 (متوسط) درصد، مجموع آگاهی 02/61 و 8/68 (متوسط) درصد، رضایتمندی 62/58 و 3/49 (خوب) درصد، نگرش 5/67 و 3/57 (متوسط) درصد و عملکرد 80/74 و 1/75 (خوب) درصد بود. افزایش میزان نگرش باعث افزایش معنادار همه متغیر های؛ آگاهی، رضایتمندی، رفتار، تعداد کلاس های شرکت شده، سن و سابقه کار شده بود.   نتیجه گیری: با توجه به مدل ارزشیابی کرک پاتریک میزان اثر بخشی دوره های آموزشی صورت گرفته در هر یک از حیطه ها در سطح « متوس ط» و « خوب » قرار گرفته است و همچنان لزوم ارتقاء سطح فرآیند آموزش و یادگیری در هر یک از سطوح آموزشی ضروری به نظر می رسد.   .

Title: بر آورد تابع تقاضای دارو در ایران از طریق بودجه خانوار در طی سالهای 1389-1370

http://tbj.ssu.ac.ir/article-1-700-fa.html

مقدمه: افزایش روزافزون مخارج دارویی از جمله نگرانی های سیاست گذاران سلامت در دنیاست. هدف پژوهش حاضر، تخمین تابع تقاضای دارو در جامعه شهری ایران به منظور کمک به سیاست گذاری مؤثر برای منطقی کردن مصرف دارو در کشور بود.   روش بررسی: پژوهش حاضر یک مطالعه توصیفی، تحلیلی است که به صورت تداومی و گذشته نگر انجام شده است. جامعه پژوهش شامل کل خانوارهای شهری کشور بوده و نمونه گیری انجام نشده است. داده های مورد نیاز از گزارش تفصیلی آمارگیری از هزینه و درآمد خانوارهای شهری و هزینه داروئی آن ها در یک دوره زمانی 20 ساله از سال 1370 تا 1389 که توسط مرکز آمار ایران، بانک مرکزی جمهوری اسلامی ایران و معاونت غذا و داروی وزارت بهداشت، درمان و آموزش پزشکی منتشر شده و با استفاده از فرم های طراحی شده توسط پژوهشگر استخراج شد. تحلیل داده ها با استفاده از نرم افزار اقتصادسنجی E-Views انجام شد. همچنین برای برآورد کشش های مختلف (کشش قیمتی و کشش درآمدی) از تحلیل رگرسیون استفاده شد .   یافته ها: یافته های پژوهش نشان می دهد که هزینه داروئی خانوارهای شهری ایران در تابع تقاضا، متغیری درون زا بوده و تابع متغیرهای شاخص قیمت دارو، درآمد کل خانوار و هزینه ویزیت پزشک می باشد. کشش قیمتی تقاضای دارو، 56/0، کشش درآمدی تقاضا، 42/0 و کشش متقاطع تقاضای دارو نسبت به هزینه ویزیت پزشک در دوره مطالعه، 18/0 برآورد گردید. نتیجه گیری: کشش پذیری کم قیمتی تقاضای دارو بدان معنی است که دارو در جامعه شهری ایران، نقش کالای ضروری را داشته و تقاضای آن حساسیت چندانی نسبت به تغییر قیمت ندارد. لذا، سیاست های کنترل مصرف دارو در کشور باید بر اقدامات غیرقیمتی نظیر افزایش آگاهی عمومی در مورد مصرف دارو و اصلاح رفتار تجویز داروی ارائه دهندگان مراقبت متمرکز شوند

Title: بررسی میزان تماس قالیبافان با گردوغبار کلی و ارتباط آن با پارامترهای عملکرد ریوی

http://tbj.ssu.ac.ir/article-1-701-fa.html

مقدمه : تماس شغلی با غبارات ایجاد شده در فرایند فرش بافی می تواند منجر به عوارض ریوی در قالیبافان گردد. هدف از این مطالعه بررسی میزان تماس قالیبافان با گردوغبار کلی و ارتباط آن با پارامترهای عملکرد ریوی می باشد.   روش بررسی : این مطالعه مورد شاهدی گذشته نگر بر روی190نفر( 95 قالیباف شهر خمین به عنوان گروه موردو 95نفر غیر قالیباف به عنوان گروه شاهدصورت پذیرفت. میزان تماس استنشاقی قالیبافان با گردوغبار کلی با استفاده از نمونه برداری هوا و روش وزن سنجی تعیین گردید. پارامتر های اسپیرومتری از جمله FVC و 1 FEV و PEF و 75-25 FEF مربوط به عملکرد ریوی قالیبافان و همچنین پارامتر های مربوط از جمله درجه حرارت محیط کار و رطوبت در محیط کار نیز جمع آوری گردید. همبستگی یافته های تماس با گردوغبار کلی با پارامتر های عملکرد ریوی مورد بررسی قرار گرفت.   یافته ها : نتایج نشان داد که میزان غلظت گردوغبار کلی در گروه مورد مطالعه برابر 47/2 ± 69/4 میلیگرم در مترمکعب بود که در مقایسه با گروه شاهد (9 33/1 ± 89/2 میلیگرم در مترمکعب) به طور معنی داری بالاتر بود ( 0001/0 P = ). در بررسی مقایسه غلظت گردوغبار کلی و عملکردهای ریوی نیز ارتباط معنی داری بین این دو پارامتر مشاهده نگردید (321/0 P = ). بررسی پارامتر های محیطی از قبیل رطوبت، درجه حرارت و نوع تهویه در کارگاه های مورد مطالعه نشان داد که با تغییرات درجه حرارت، رطوبت و تهویه کارگاه می تواند بر روی عملکرهای ریوی تاثیرگذار باشد. بررسی ها نشان داد که رابطه معکوسی بین سابقه کاری و شاخص های FVC و FEV و PEF و 75-25 FEF وجود داشته و با افزایش سابقه کاری این شاخص ها کاهش پیدا می کنند. نتیجه گیری : نتایج این مطالعه نشان می دهد که با وجود اجرای طرح بقا در مناطق مورد مطالعه، تماس قالیبافان با گردوغبار کلی می تواند به عنوان یک عامل خطرناک شغلی به میزان بالایی وجود داشته باشد.

Title: بهبود سامانه هضم در آزمون اکسیژن مورد نیاز شیمیایی به کمک نانو ذرات نقره به منظور سنجش بارآلی فاضلاب صنایع داروسازی

http://tbj.ssu.ac.ir/article-1-704-fa.html

مقدمه: سنجش دقیق بار آلی یکی از پارامتر های مهم و تعیین کننده در انتخاب طراحی فرایند تصفیه مناسب برای فاضلاب های خانگی و صنعتی به شمار می رود. انجام آزمون اکسیژن خواهی شیمیایی در نمونه های حقیقی همواره با تداخل عوامل مزاحم، هضم ناقص نمونه و مصرف بالای مواد آلاینده محیط زیست روبرو بوده است. هدف از انجام این مطالعه کاربردی- تحقیقی بهبود سامانه هضم در آزمون اکسیژن مورد نیاز شیمیایی به کمک نانوذرات نقره به منظور سنجش بارآلی پساب صنایع داروسازی است.   روش بررسی: در این پژوهش بجای استفاده از سولفات نقره، از نانو ذرات نقره با اندازه متوسط 20 نانومتر در آزمون اکسیژن مورد نیاز شیمیایی استفاده شد و پس از بهینه سازی متغیرهایی همچون دما و زمان هضم و تعیین میزان بهینه کاتالیست، کلیه اعداد شایستگی برای روش پیشنهادی مشخص شد و در شرایط بهینه بار آلی پساب کارخانه داروسازی مشتمل بر فاضلاب بهداشتی و صنعتی و فاضلاب مخلوط اندازه گیری شد و کلیه نتایج با روش مرجع مورد مقایسه قرار گرفت.   یافته ها: نتایج این بررسی نشان می دهد که تنها با بکارگیری 16 درصد از جرم نقره بکاررفته در روش مرجع می توان به کارایی موثری از نظر هضم نمونه های فاضلاب داروسازی و برخی ترکیبات شیمیایی دیرهضم دست یافت. نتایج بررسی، حداقل دما و زمان لازم برای دستیابی به شرایط بهینه هضم را نشان می دهد که در حضور مقادیر اندک نانو ذرات نقره، عملیات هضم در زمان 120 دقیقه در دمای بهینه 150 درجه سانتی گراد کامل می شود. همچنین روش پیشنهادی از میانگین انحراف استاندارد 03/4 و درصد خطای 06/5 برخوردار است(12= n ) که در مقایسه با روش استاندارد از دقت و صحت بالاتری برخوردار است. مقادیر حد تشخیص و حداندازه گیری به ترتیب 5 و 15 میلی گرم در لیتر بودند و این سیستم از دامنه خطی 15 تا 300 میلی گرم در لیتر برخوردار است.   نتیجه گیری : باتوجه به کارایی بسیار خوب سامانه بر هضم مواد آلی در حضور نانو ذرات نقره، در تعیین بار آلی نمونه های فاضلاب صنایع داروسازی و برخورداری از تکرارپذیری بالا، مطابقت داده ها با روش مرجع، دامنه خطی گسترده، بکارگیری مقادیر بسیار کمتر نقره و سازگاری بیشتر با محیط زیست استفاده از این روش برای سنجش بار آلی فاضلاب صنایع داروسازی و فاضلاب سایر صنایع مشابه پیشنهاد می شود.

Title: بررسی وضعیت تغذیه انحصاری با شیر مادر تا 6 ماهگی و برخی عوامل مرتبط با آن در کودکان 12-6 ماهه تحت پوشش مراکز بهداشتی درمانی شهری شهر اردکان

http://tbj.ssu.ac.ir/article-1-1812-fa.html

مقدمه: تغذیه انحصاری با شیر مادر تا 6 ماهگی بهترین تغذیه برای کودک می باشد که تأمین کننده نیازهای جسمی، فیزیو لوژیک و روحی او تا شش ماهگی است و باعث کاهش بروز برخی عفونت ها می شود و تابحال کمتردر تحقیقات بهداشتی مورد توجه قرار گرفته است. لذا مطالعه  حاضر با هدف تعیین شیوع تغذیه انحصاری با شیر مادر تا شش ماهگی و برخی عوامل مرتبط با آن در شهر اردکان ارائه می شود. روش بررسی: طی یک مطالعه توصیفی مقطعی در مرداد ماه 1390،  413 کودک 12- 6 ماهه از 4 مرکز بهداشتی درمانی شهری شهر اردکان بصورت سر شماری مورد بررسی قرار گرفتند. اطلاعات بوسیله پرسشنامه محقق ساخته و مصاحبه با مادر جمع آوری گردید. پرسشنامه شامل اطلاعات دموگرافیک و نیز اطلاعات مربوط به وضعیت تغذیه کودک در 6 ماهه اول زندگی بود. تمامی مادران داوطلبانه در مطالعه  شرکت کردند. یافته ها: 8/50 %اطفال مورد بررسی  پسر و 2/49% دختر بودند میزان تغذیه انحصاری فقط با شیر مادر تا 6 ماهگی 1/6% درصد و میزان تغذیه انحصاری با شیر مادر و آب جوشیده 6/34% بود. میانگین مدت تغذیه انحصاری فقط با شیر مادر22/2±49/3 ماه و میانگین مدت تغذیه انحصاری با شیر مادر و آب جوشیده           79/1± 55/4 ماه بود. بین تغذیه انحصاری با شیر مادر و توقف وافت رشد ارتباط معنی دار آماری وجود داشت. مهم ترین موانع تغذیه انحصاری با شیر مادر از دید مادران بترتیب گریه بدون علت و بیقراری نوزاد ودرک ذهنی از ناکافی بودن شیر مادر گزارش گردید. نتیجه گیری: میزان تغذیه انحصاری با شیر مادر در سطح پایین گزارش شد و اکثر مادران شیرده در زمینه کافی بودن شیر خود در شش ماهه اول جهت تغذیه شیر خوار توجیه نبوده و  علت عدم موفقیت خود را در انجام این رفتار موارد نامربوطی از جمله بیقراری نوزاد ودرک ذهنی از کافی نبودن شیر ذکر کردند که نشان دهنده عدم آگاهی لازم  آنان در این زمینه می باشد، بعلاوه افراد با سواد در امر تغذیه انحصاری با شیر مادر موفق تر از افراد کم سواد بودند که همه موارد فوق گواه برنیاز آموزشی بیشتر جهت افزایش آگاهی مادران شیرده در این زمینه میباشد.

Title: بررسی سطح حساسیت سوسری آلمانی نسبت به حشره کش های گروه کاربامات و فسفره به روش In vivo

http://tbj.ssu.ac.ir/article-1-706-fa.html

مقدمه: سوسری آلمانی ناقل مکانیکی عوامل بیماریزای مختلف از قبیل ویروسها، باکتریها، تک یاخته ها و تخم انگلها می باشد. هدف از این مطالعه، تعیین سطح حساسیت سوسری آلمانی نسبت به حشره کش های بندیوکارب و کاربایل از گروه کاربامات، مالاتیون و کلرپیریفوس از گروه فسفره، به روش In vivo می باشد.   روش بررسی: در این مطالعه سوشهای وحشی سوسری آلمانی پس از جمع آوری از چند بیمارستان و خوابگاه در شهر تهران و انتقال به انسکتاریوم تحت شرایط یکسان آزمایشگاهی پرورش داده شدند و سطح حساسیت آنها با استفاده از غلظتهای تمایزی تعیین شده بر روی سوش حساس نسبت به حشره کش های بندیوکارب، کاربایل، مالاتیون و کلرپیریفوس به روش In vivo مورد بررسی قرار گرفت.   یافته ها: نتایج حاصل از اثر غلظتهای تمایزی بر روی سوشهای وحشی سوسری آلمانی نشان داد که پائین ترین غلظت تمایزی به حشره کش کلرپیریفوس ( mg/m2 63/6 ) و بالاترین آن به حشره کش کارباریل ( mg/m2 11/390) اختصاص داشت. اکثر سوشهای وحشی درجاتی از مقاومت و تحمل را نسبت به حشره کشهای بندیوکارب، کارباریل، مالاتیون و کلرپیریفوس از خود نشان دادند.   بحث و نتیجه گیری: نتایج این مطالعه نشان می دهد که حشره کش های گروه کاربامات بخصوص کارباریل در حال حاضر برای مبارزه با سوسری آلمانی کارآیی ندارند و توصیه می شود مصرف این حشره کشها متوقف گردد. در مورد حشره کش های گروه فسفره نیز توصیه می شود به دلیل مقاومت به مالاتیون و احتمال وجود مقاومت متقاطع ( Cross resistance ) بین ترکیبات مختلف این گروه از مصرف حشره کشها خودداری گردد.

Title: بررسی کارایی نانوذرات آلومینای اصلاح شده با لیگاند شیف‌باز در حذف کروم شش ظرفیتی از محلول آبی در شرایط اسیدی

http://tbj.ssu.ac.ir/article-1-707-fa.html

مقدمه: فلزات سنگین از جمله آلاینده­های مهمی هستند که امروزه غلظت آن­ها در محیط زیست با گسترش شهرها و توسعه صنایع افزایش یافته است. یکی از مهمترین فلزات سنگین، کروم است که به صورت اکسیدهای سه و شش ظرفیتی در صنایعی مانند آبکاری، شیشه، سرامیک، دباغی و چرم کاربرد دارد. کروم شش ظرفیتی دارای مخاطرات بهداشتی اثبات شده ای مانند سرطان­زایی است. هدف از این مطالعه بررسی کارایی نانوذرات آلومینای اصلاح شده با لیگاند شیف باز در حذف کروم شش ظرفیتی از محلول­های آبی در شرایط اسیدی است.   روش بررسی: این تحقیق یک مطالعه تجربی است که در آن آزمایشات در شرایط ناپیوسته و در دمای C ° 20 انجام شده و اثر غلظت اولیه کروم، جرم جاذب و زمان واکنش بر حذف کروم شش ظرفیتی توسط جاذب نانوذرات آلومینا مورد بررسی قرار گرفت. برای تهیه جاذب، نانوذرات آلومینا از شرکت نانوکیمیا پژوه تهیه و برای افزایش ظرفیت جذب، با لیگاند شیف­باز و در حضور سورفاکتانت مناسب اصلاح شد. مطالعه روی نمونه­های سنتزی حاوی غلظت های 2و 5 میلی­گرم در لیتر کروم انجام گرفت. غلظت مقادیر مجهول کروم شش ظرفیتی به روش اسپکتروفتومتری در حضور معرف 1و5-دی فنیل کاربازاید و طول موج 540 نانومتر تعیین شد.   یافته ها: با توجه به نتایج، با افزایش جرم جاذب و زمان واکنش مقدار حذف آلاینده افزایش و با افزایش غلظت اولیه کروم مقدار حذف کاهش یافت. داده­های این تحقیق نشان داد که بیشترین میزان جذب کروم شش ظرفیتی توسط نانوذرات آلومینای اصلاح شده با لیگاند شیف­باز در مدت زمان 180 دقیقه انجام و آزمایش به حالت تعادل می­رسد. بیشترین میزان راندمان حذف و ظرفیت جذب در شرایط بهینه(جرم جاذب 1/0 میلی­­گرم در لیتر و3 pH = )، برای غلظت اولیه کروم 5 میلی­گرم در لیتر و زمان 180 دقیقه به­ترتیب برابر با 4/83 درصد و mg/g 17/4 می­باشد.   نتیجه گیری: نانوذرات آلومینای اصلاح شده با لیگاند شیف ­ باز جاذبی مؤثر در حذف کروم شش ظرفیتی از محلول­های آبی می­باشد.

Title: تعیین مقدار فلزات سنگین در برخی نمونه های پر مصرف سیگار و توتون های معطر موجود در بازار ایران

http://tbj.ssu.ac.ir/article-1-1813-fa.html

چکیده مقدمه: به منظور پیشگیری از اثرات مضر فلزات سنگین بر سلامت انسانها، بررسی مقدار فلزات سنگین در سیگارها وتوتون های معطر امری ضروری است، لذا این پژوهش با هدف تعیین مقدار فلزات سنگین در برخی نمونه های پر مصرف سیگار و توتون های معطر موجود در بازار ایران انجام گرفته است. روش بررسی: در این مطالعه 9 نمونه سیگار و 3 نمونه توتون معطر پر مصرف و موجود در بازار ایران انتخاب و از نظر مقدار فلزات سنگین سرب، روی، کادمیوم، کروم، نیکل، مس و آهن در توتون آنها بررسی شدند. فلزات سنگین نمونه ها با روش هضم اسیدی و دستگاه جذب اتمی شعله ای قرائت گردید. برای اطمینان از صحت نتایج، آزمایشات برای دو سری نمونه بصورت متوالی انجام شد. یافته ها: میانگین غلظت فلز آهن با حداکثر µg/g 28/78± 45/473 در تمامی نمونه های سیگار بالاترین مقدار را بخود اختصاص داد. در حالی که کمترین میانگین غلظت فلز مربوط به کروم با مقدار µg/g 03/0 ± 33/1 بود. آزمون آماری بین میانگین مقادیر فلزات سنگین در سیگارها و توتون های ایرانی و خارجی اختلاف معنی داری نشان نداد(05/0P > ). نتیجه گیری: در مجموع با توجه به نتایج به دست آمده وجود فلزات سنگین در برخی نمونه های پر مصرف سیگار و توتون های معطر موجود در بازار ایران مشخص شد. لذا باتوجه به اهمیت این موضوع، برای حفظ سلامت انسان باید تلاش جدی صورت گیرد.

Title: مقایسه فرایند های ارزیابی کنارآمدن و ارزیابی تهدید در پیش بینی قصد و رفتارتغذیه ای پیشگیری کننده از سرطان در مادران مراجعه کننده به مراکز بهداشتی- درمانی شهر یزد

http://tbj.ssu.ac.ir/article-1-709-fa.html

مقدمه: فرایند میانجی شناختی ارزیابی تهدید در تئوری انگیزش محافظت رفتارهای ناسازگارانه را ارزیابی می کند و سازه های آن شامل حساسیت، شدت و پاداش های درک شده درونی و بیرونی می باشد. فرایند میانجی شناختی ارزیابی کنارآمدن که توانایی مقابله و دفع خطر تهدید شده را ارزیابی می کند، شامل سازه های خودکارآمدی، کارآمدی پاسخ و هزینه درک شده می باشند هدف مطالعه حاضر مقایسه ارزیابی کنارآمدن و ارزیابی تهدید در پیش بینی قصد و رفتار تغذیه ای پیشگیری کننده از سرطان در مادران شهر یزد می باشد.   روش بررسی: در این مطالعه توصیفی تحلیلی به روش مقطعی، 220 نفر از مادران مراجعه کننده به مراکز بهداشتی درمانی شهر یزد مورد مطالعه قرار گرفتند. این افراد به روش تصادفی چند مرحله ای انتخاب شدند. ابزار جمع آوری اطلاعات، پرسشنامه ای با روایی و پایایی قابل قبول بود. پرسشنامه شامل اطلاعات دموگرافیک، سازه های فرآیند ارزیابی کنارآمدن و تهدید، به انضمام قصد و رفتار تغذیه ای پیشگیری کننده از سرطان بود که به صورت خود گزارش دهی تکمیل گردید. تجزیه و تحلیل داده ها با کمک نرم افزار16 spss و آزمونهای آماری ANOVA ، ضریب همبستگی و Linear regression analysis انجام شد.   یافته ها: یافته های این پژوهش نشان داد که فرایند ارزیابی تهدید با قصد و رفتار تغذیه ای پیشگیری کننده از سرطان همبستگی منفی و فرایند ارزیابی کنار آمدن با قصد و رفتار تغذیه ای پیشگیری کننده از سرطان همبستگی مثبت و معناداری در سطح01/0 داشتند. میزان پیش بینی قصد رفتار تغذیه ای پیشگیری کننده از سرطان توسط ارزیابی تهدید و ارزیابی کنارآمدن 4/28% بود که تنها فرآیند ارزیابی کنارآمدن پیش بینی کننده معناداری بودند. (05/0 > p )   نتیجه گیری: مطالعه حاضر نشان می دهد که فرآیند میانجی شناختی ارزیابی کنارآمدن در پیش بینی قصد و رفتار تغذیه ای پیشگیری کننده از سرطان موفق تر بوده است، لذا با کاستن هزینه درک شده و بالا بردن کارآمدی پاسخ و خودکارآمدی با هدف تسهیل پذیرش رفتارتغذیه ای مناسب می تواند به عنوان یک اصل در آموزش مورد توجه قرار گیرد.

Title: میزان پذیرش طرح توزیع شیر رایگان و برخی عوامل موثر بر آن در دبیرستان های دخترانه شهر یزد

http://tbj.ssu.ac.ir/article-1-710-fa.html

مقدمه: با توجه به اهمیت مصرف لبنیات در دوره نوجوانی جهت رشد مطلوب، توزیع شیر رایگان توسط آموزش و پرورش در سال های اخیر در مدارس کشور اجرا شده است. این مطالعه جهت تعیین میزان پذیرش این طرح و عوامل موثر بر آن در دبیرستان های دخترانه شهر یزد انجام گرفت.   روش بررسی: این مطالعه توصیفی- مقطعی در سال 1389 با مشارکت 703 دانش آموز دختر دبیرستانی انجام گرفت. نمونه گیری به صورت تصادفی طبقه ای از هر دو منطقه آموزش و پرورش صورت گرفت. جمع آوری داده ها با استفاده از پرسشنامه حاوی سوالات زمینه ای، سوالاتی در مورد میزان پذیرش شیر و عوامل موثر بر آن در بین دانش آموزان انجام گرفت و داده ها با استفاده از نرم افزار SPSS تجزیه شد.   یافته ها: 3/37 % از دانش آموزان اصلا شیرهای توزیع شده را مصرف نمی کردند. بین میزان پذیرش شیرهای توزیعی و نمره آگاهی ارتباط معنی داری مشاهده نشد ولی میزان پذیرش با نمره نگرش دانش آموزان ارتباط معنی داری داشت. از بین دانش آموزانی که شیرهای توزیع شده را مصرف می کردند 63% علت مصرف خود را مفید بودن شیر و از بین کسانی که این شیرها را مصرف نمی کردند 64% علت عدم مصرف خود را خوش طعم نبودن شیرهای ارائه شده ذکر کردند.   نتیجه گیری: میزان پذیرش شیرهای توزیع شده در حد مطلوب نبوده و با توجه به نتایج فراوانترین دلیل عدم پذیرش، طعم نامطلوب بوده است. بهبود کیفیت و حتی اصلاح طعم شیرهای این طرح در افزایش پذیرش نقش به سزایی خواهد داشت.

Title: شکایت از درد در اندام فوقانی و بررسی رابطه آن با استرس شغلی در کارکنان اداری

http://tbj.ssu.ac.ir/article-1-711-fa.html

مقدمه : بدون شک استرس شغلی و اختلالات اسکلتی-عضلانی رابطه مستقیمی با سلامت کارکنان دارد. بنا به گزارش معاونت درمان سازمان تأمین اجتماعی بیماری‌های اسکلتی – عضلانی علت4/14درصد از کارافتادگی‌های کلی در ایران بوده، همچنین بیشترین تعداد مراجعات به کمیسیون‌های پزشکی بدوی سازمان تأمین اجتماعی به علت ناراحتی‌های اسکلتی - عضلانی بوده است. هدف از این مطالعه تعیین میزان شکایت از درد در اندام فوقانی و بررسی رابطه آن با استرس شغلی در کارکنان اداری می‌باشد.   روش بررسی : این یک مطالعه مقطعی می باشد که در سال 1390 انجام گرفت. نمونه در این مطالعه 320 نفر از کارکنان اداری دانشگاه علوم پزشکی شهید بهشتی و دانشگاه علوم بهزیستی و توان‌بخشی می‌باشند. که 37 درصد نمونه را مردان و 63 درصد را زنان تشکیل می‌دهند. ابزار مورد استفاده در این پژوهش چارت نشان دهنده‌ی اختلالات اسکلتی عضلانی BDC ، پرسشنامه استرس شغلی HSE و همچنین یک پرسشنامه باز پاسخ می‌باشد. روش نمونه گیری طبقه ای بوده که بر طبق جمعیت هر دانشگاه تعداد نمونه انتخاب می‌شدند،و از نرم افزار SPSS نسخه 16برای آنالیز آماری استفاده شد.   یافته ها : نتایج نشان داده بیشترین اختلالات اسکلتی عضلانی مربوط به کمر با 2/28 درصد و کمترین آن مربوط به بازو و ساعد با7/4درصدمی‌باشد. نتایج همچنین نشان داد که شکایت از درد در کمر، بازو، گردن و شانه اختلاف معنی داری بین زن و مرد دارد.همچنین میزان استرس بالا، 11درصد می‌باشد. در زیر مقیاس مربوط به استرس بیشترین میزان استرس بالا مربوط به روابط بین فردی 1/73 درصد و کمترین مقدار آن مربوط به کنترل با 5/2درصد می‌باشد. همچنین هیچ رابطه معناداری بین شکایت از درد اندام فوقانی با زیر مقیاس‌های استرس شغلی وجود نداشت.   نتیجه گیری : نتایج این مطالعه نشان داد که میزان استرس شغلی و درد در اندام فوقانی در نمونه مورد نظر بالا می‌باشد اما رابطه معنی داری بین درد در اندام فوقانی و استرس شغلی پیدا نشد، با توجه به میزان بالای استرس و اختلالات اسکلتی عضلانی در این نمونه و با توجه به اینکه مطالعات گذشته، نقش استرس و اختلالات اسکلتی عضلانی را در بهره‌وری، غیبت از کار و رضایت شغلی را به اثبات رسانیده‌اند پیشگیری از این عوامل ضروری می‌باشد.    .

Title: بررسی اثر بلانکیت خوراکی بر قند خون ناشتای موش بزرگ آزمایشگاهی

http://tbj.ssu.ac.ir/article-1-712-fa.html

مقدمه: دیابت یکی از شایعترین بیماریهای متابولیکی و چهارمین علت مرگ و میر در جهان می باشد. و با نقص در متابولیسم کربوهیدراتها، چربیها وپروتئین ها همراه با احتمال کمبود ترشح انسولین با درجات متفاوتی از مقاومت به انسولین مشخص می شود . استان یزد از دیرباز به شهر شیرینی معروف بوده و متاسفانه در زمینه دیابت رکورددار است میزان شیوع بیماری دیابت در استان ‪ یزد ۱/۵تا دو برابر بیش از متوسط کشوری آن است. میزان شیوع این بیماری در استان یزد ۴/۷ درصد و درکشور ۲/۳درصد است بررسی های انجام شده، نشان دهنده بالا بودن بیش از حد مصرف بلانکیت( Na2So2O4 ) به عنوان سفیدکننده می باشد. به همین دلیل بر آن شدیم تا اثر دیابت زائی بلانکیت را بررسی کنیم.   روش بررسی : این مطالعه مداخله ای in vivo در حیوان آزمایشگاهی ، از نوع کنترل دار بر روی موش بزرگ آزمایشگاهی انجام گردید در ابتدا30 موش بزرگ سفید آزمایشگاهی نربا وزن بین 250-200 گرم به طور تصادفی به 5گروه 6 تایی تقسیم کرده. با استفاده از لوله موئینه هپارینه از گوشه چشم آنها خون گرفته شده سپس گروه کنترل آب و در گروه مورد بلانکیت با غلظت های ppm 4، 20، 100، 200 به آب آنها اضافه نموده و در روزهای 15، 30 ،90 ازگوشه چشم آنها خون گیری مجدد شد و میزان غلظت قند خون آنها ( FBS ) تعیین گردید و پس از آن اطلاعات آنها جمع آوری و با استفاده از آزمونهای ANOVA و t-test موردتجزیه وتحلیل آماری قرار گرفت.   یافته ها :نتایج بدست آمده نشان داده است که سطح قند خون دراثر تجویز بلانکیت افزایش یافته و به غلظت نیز وابسته می باشد که بیشترین افزایش مربوط به دوز 200 میلی گرم بر کیلوگرم می باشد(001/0 PV= ).   بحث ونتیجه گیری : نتایج این مطالعه نشان می دهد که بلانکیت می تواند در موش باعث افزایش قند شود. لذا پیشنهاد می شود دراین زمینه پژوهشهایی از جمله بررسی مقاومت به انسولین، میزان ترشح انسولین و .... انجام گیرد. تا جواب گوی این سوال باشد که آیا بلانکیت باعث بیماری دیابت می گردد یا خیر؟

Title: اثر محیط کشت بر روی رشد سلولی، کمیت و کیفیت بیوماس در میکروجلبک نانوکلروپسیس اوکولاتا جهت تولید بیودیزل (سوخت سبز)

http://tbj.ssu.ac.ir/article-1-714-fa.html

مقدمه: نگرانی در مورد بحران انرژی همراه با تغییرات آب و هوایی و افزایش گرمایش جهانی، باعث توجه بیشتر به تولید انرژی بیودیزل از میکروجلبکها شده است. رشد سلولی، تولید بیوماس و تجمع لیپید در میکروجلبک از عوامل تعیین کننده بازدهی اقتصادی بیودیزل بوده و هدف از این مطالعه ، بررسی نقش محیط کشت در افزایش بازدهی عوامل مذکور می باشد.   روش بررسی: میکروجلبک نانوکلروپسیس اوکولاتا درچهار محیط Walne ، F/2 ، Sato و TMRL کشت داده شد. میزان رشد سلولی به طور روزانه از طریق اندازه گیری دانسیته سلولی با استفاده از اسپکتوفتومتر تعیین گردید. تولید بیوماس و تجمع لیپید در دو فاز رشد لگاریتمی و رشد ثابت میکروجلبک، اندازه گیری و ترکیبات تشکیل دهنده بوسیله گازکروماتوگرافی تشخیص داده شد. مطابق استاندارد، کیفیت روغن استخراج شده از هر محیط کشت جهت تولید بیودیزل ، بوسیله اندیس ید تعیین گردید.   یافته ها: نتایج حاصل نشان داد بیشترین میزان رشد سلولی و تولید بیوماس در محیط کشت Walne و به ترتیب برابر day-1 2616/0 و gl-1 652/2 می باشد . بیشترین درصد تبدیل بیوماس به لیپید نیز در محیط کشت TMRL و برابر 22/37 درصد وزن خشک سلولی حاصل شد. بیشترین بازدهی لیپید مربوط به محیط کشت Walne و برابر gl-1day-1 1057/0بود و بیشترین مقدار اسیدهای چرب غیر اشباع چندگانه PUFA ( Polyunsaturated Fatty Acids )در محیط کشت Walne و در مرحله رشد لگاریتمی و برابر 99/42 درصد اسید چرب بدست آمد. بیشترین مقدار تری گلیسرید نیز در مرحله رشد ثابت و در محیط کشت sato برابر 25/75 درصد اسید چرب بدست آمد .   نتیجه گیری: میکروجلبک نانوکلروپسیس اوکولاتا ، حداکثر بازدهی جهت مقاصد آبزی پروری و مصارف انسانی و همچنین تولید بیودیزل را به ترتیب در فاز رشد لگاریتمی و رشد ثابت و در محیط کشت Walne نشان داد.

Title: مقایسه تاثیر آموزش بهداشت دهان و دندان ارائه شده توسط رابطین بهداشت و انترن دندانپزشکی طرح جامعه نگر بر آگاهی، نگرش و عملکرد زنان خانه دار

http://tbj.ssu.ac.ir/article-1-715-fa.html

مقدمه: دندانپزشکی جامعه نگر یکی از راهبردهای اساسی جهت ارتقای سطح سلامت دهان در جامعه محسوب می شود. این رویکرد اساساً بر دندانپزشکی پیشگیری و آموزش بهداشت با جلب مشارکت مردم جامعه تاکید دارد. رابطین بهداشت زنان داوطلبی هستند که از سال 1369 به صورت آزمایشی و از سال 1372 به صورت فراگیر با هدف ارتقاء سلامت با مراکز بهداشتی- درمانی همکاری می نمایند. هدف از این مطالعه مقایسه تاثیر آموزش بهداشت دهان و دندان ارائه شده توسط رابطین بهداشت و انترن دندانپزشکی(انترن سال اخر که تمام واحد های درسی دوره عمومی را گذرانده است) طرح جامعه نگر بر آگاهی، نگرش و عملکرد زنان خانه دار بوده است.   روش بررسی: این مطالعه یک مطالعه نیمه تجربی است.در این مطالعه ابتدا جزوه آموزشی و پرسشنامه طراحی شد. سپس رابطین بهداشت تحت آموزش قرار گرفتند. در مرحله بعد 80 نفر از زنان خانه دار مراجعه کننده به مراکز بهداشتی- درمانی اکبری (صفائیه)، مرکز بهداشتی درمانی شهید دکتر رسول فرشاد با روش نمونه گیری آسان انتخاب شدند. شرکت کنندگان در این مطالعه به 2 گروه 40 نفره ( A و B ) (گروه A ‌ گروه تحت نظرانترن دندانپزشکی وگروه B گروه تحت نظر رابطین بهداشت) تقسیم شدند. زنان هر 2 گروه معاینه شدند و شاخص پلاک آنها محاسبه شده و سپس پرسشنامه ها را تکمیل نمودند. گروه A توسط انترن دندانپزشکی و گروه B توسط رابطین بهداشت تحت آموزش قرار گرفتند. سه ماه بعد نیز مجددا پرسشنامه ها تکمیل شد و شاخص پلاک زنان خانه دار بررسی شد. داده ها با استفاده از تست های آماری Wilcoxon Signed Ranks و Mann-Whitney آنالیز شدند(05/0 P< ).   یافته ها: نتایج به دست آمده از این مطالعه نشان می دهد که بعد از آموزش بهداشت سطح آگاهی، نگرش و عملکرد در کل نمونه ها و در گروه A و B به صورت معنی داری افزایش یافت. لازم به ذکر است که شاخص پلاک در هر دو گروه کاهش معنی داری نشان داد، اما اختلاف معنی داری بین آموزش توسط انترن دندانپزشکی و رابطین بهداشت دیده نشد.   نتیجه گیری: نتایج به دست آمده نشان می دهد که تفاوت چندانی در نتایج بعد از آموزش ارائه شده توسط انترن دندانپزشکی و رابطین بهداشت دیده نمی شود. این مسئله می تواند تقویت کننده فرضیه مناسب بودن رابطین بهداشت جهت انجام آموزش های سلامت دهان باشد.

Title: بررسی ابعاد ساختارسازمانی در بیمارستان های عمومی شهرستان یزد با رویکرد مکانیک –

http://tbj.ssu.ac.ir/article-1-716-fa.html

مقدمه : نخستین مسئله ای که هنگام طرح ریزی یک سازمان مطرح می شود،مسئله ساختار است .در بین ابعاد ساختاری ،پیچیدگی،رسمیت و تمرکز نسبت به سایر ابعاد ساختاری در ایجاد ساختار سازمانی و اداره موثر و اثربخش سازمان نقش مهم تری دارند و آن ها را ابعاد اصلی می نامند. هدف از انجام این پژوهش ، بررسی ابعاد (پیچیدگی ، رسمیت و تمرکز) در بیمارستان های عمومی جامعه مورد پژوهش و تعیین وضعیت موجود ابعاد ساختاری و تعیین وضعیت مطلوب ساختار با رویکرد مکانیک - ارگانیک و ارائه الگوی مناسب ساختار می باشد.   روش بررسی : در این پژوهش تعداد 263 نفر از کارکنان 8 بیمارستان عمومی شهرستان یزد با استفاده از شیوه نمونه گیری تصادفی طبقه ای و سرشماری مورد پرسش واقع شدند. در این مطالعه از پرسشنامه های استاندارد طراحی ساختار مطلوب "ویگا و یانوزاس" وپرسشنامه ارزیابی ابعاد ساختاری (پیچیدگی، رسمیت و تمرکز) "استیفن رابینز" به عنوان ابزار پژوهش استفاده شد . اطلاعات حاصل به دو شیوه توصیفی ( میانگین ، انحراف معیار و درصد فراوانی ) و تحلیلی با استفاده از آزمون دو جمله ای و آنالیز واریانس با نسخه 19 نرم افزار SPSS ، تحلیل گردید   یافته ها : این مطالعه نشان داد میزان پیچیدگی در بیمارستان های مورد مطالعه با کسب رتبه 32 نسبتا زیاد و میزان رسمیت با کسب رتبه 24 بالاتر از حد متوسط و میزان تمرکز با کسب رتبه 30 در حد متوسط می باشد و ساختار مطلوب از نظر پاسخگویان، ساختارمکانیکی است(001/0= P ).   نتیجه گیری : با توجه به تصریح پرسش شوندگان در خصوص اتخاذ ساختار بسیار مکانیکی به عنوان ساختار مطلوب، ساختار منتج از این الگو را ساختاری بلند تشکیل می دهد. در این ساختار مکانیکی، امور با پیچیدگی بالا، رسمیت نسبتا زیاد و تمرکز زیاد، انجام می گیرد. هرچند بر اساس الگوی پیشنهادی می توان با تغییر در اجزاء ابعاد پیچیدگی،رسمیت و تمرکز و افزایش میزان آن به ساختار مطلوب دست یافت لیکن به نظر می رسد ارائه یک ساختار ثابت و خاص برای بیمارستان ممکن نیست بلکه می بایست همگام با تغییرات، ساختاراقتضائی مناسب برای بیمارستان طراحی شود.
